# Decreased Expression of Cytotoxic Proteins in Decidual CD8^+^ T Cells in Preeclampsia

**DOI:** 10.3390/biology10101037

**Published:** 2021-10-13

**Authors:** Violeta Soljic, Maja Barbaric, Martina Vukoja, Marina Curlin, Martina Orlovic Vlaho, Edita Cerni Obrdalj, Lidija Lasic Arapovic, Daniela Bevanda Glibo, Katarina Vukojevic

**Affiliations:** 1 Laboratory of Morphology, Department of Histology and Embryology, School of Medicine, University of Mostar, 88 000 Mostar, Bosnia and Herzegovina; violeta.soljic@mef.sum.ba (V.S.); maja.pivic@mef.sum.ba (M.B.); martina.vukoja@mef.sum.ba (M.V.); lidija.lasic-arapovic@mef.sum.ba (L.L.A.); 2 Faculty of Health Studies, University of Mostar, 88 000 Mostar, Bosnia and Herzegovina; marina.curlin@fzs.sum.ba (M.C.); martina.orlovic.vlaho@mef.sum.ba (M.O.V.); 3 Department of Obstetrics, Gynecology University Clinical Hospital Mostar, 88 000 Mostar, Bosnia and Herzegovina; 4 Department of Family Medicine, School of Medicine, University of Mostar, 88 000 Mostar, Bosnia and Herzegovina; edita.cerni-obrdalj@mef.sum.ba; 5 Study Program of Dental Medicine, School of Medicine, University of Mostar, 88 000 Mostar, Bosnia and Herzegovina; 6 Department of Gastroenterology, University Hospital Mostar, 88000 Mostar, Bosnia and Herzegovina; daniela.bevanda-glibo@mef.sum.ba; 7 Department of Anatomy, Histology and Embryology, School of Medicine, University of Split, Šoltanska 2, 21 000 Split, Croatia

**Keywords:** preeclampsia, perforin, granulysin, granzyme A, granzyme B, FOXP3, CD8

## Abstract

**Simple Summary:**

CD8^+^ T cells are prominent decidual cells in the third trimester of healthy human pregnancy. They have a cytotoxic capacity which may control invasion of extravillous trophoblast and therefore affect placentation and play the role in development of preeclampsia. In this study, we examined the expression of CD8^+^ T cells in decidual tissue and peripheral blood of women with severe and mild preeclampsia in comparison to gestational age-matched healthy pregnancies. Additionally, the expression of cytotoxic proteins in CD8^+^ T cells was examined in order to specify their subpopulations.

**Abstract:**

In our study, we aimed to establish expression of cytotoxic CD8^+^ T cells in the decidua basalis and the maternal peripheral blood (mPBL) of severe and mild preeclampsia (PE) and compare to healthy pregnancies. Decidual tissue and mPBL of 10 women with mild PE, 10 women with severe PE, and 20 age-matched healthy pregnancy controls were analyzed by double immunofluorescence and qPCR, respectively. By double immunofluorescence staining, we found a decreased total number of cells/mm^2^ in decidua basalis of granulysin (GNLY)^+^ (*p* ˂ 0.0001), granzyme B (GzB)^+^(*p* ˂ 0.0001), GzB^+^CD8^+^(*p* ˂ 0.0001), perforin (PRF1)^+^ (*p* ˂ 0.0001), and PRF1^+^CD8^+^ (*p* ˂ 0.01) in the severe PE compared to control group. Additionally, we noticed the trend of lower mRNA expression for GNLY, granzyme A (GZMA), GzB, and PRF1 in CD8^+^ T cells of mPBL in mild and severe PE, with the latter marker statistically decreased in severe PE (*p* ˂ 0.001). Forkhead box P3 (FOXP3) mRNA in CD8^+^ T cells mPBL was increased in mild PE (*p* ˂ 0.001) compared to controls. In conclusion, severe PE is characterized by altered expression of cytotoxic CD8^+^ T cells in decidua and mPBL, suggesting their role in pathophysiology of PE and fetal-maternal immune tolerance.

## 1. Introduction

Successful pregnancy outcome and fetal growth are highly dependent on the normal placental development and function. One of the major events during the process of placentation is invasion of extravillous trophoblasts (EVT) [1]. Incomplete and shallow invasion can lead to the development of pregnancy disorders, including intrauterine fetal growth restriction (IUGR), preterm labor, miscarriage, and, most commonly, PE [2,3]. There is no consistent and uniform classification of PE, but the one based on the severity of symptoms into mild and severe PE is commonly used [4,5,6]. Different forms of PE have significantly different clinical courses, outcomes, and, according to the latest data, pathophysiology [7,8]. However, unpredictability is one of the common features of this disease, and what is at one moment a mild disease can very easily progress to severe PE which, regardless of the form, requires constant caution [4].

In addition to EVT invasive abilities, its capacity in promoting the immune tolerance in decidua was put in the focus of interest [9]. Decidua is a place of great immune importance. It is the site of direct contact of trophoblasts with maternal immunocompetent cells [10,11,12]. Immune cells that inhabit the decidua not only maintain placental function, but control trophoblast invasion, prevent fetal rejection, and participate in defense against infections during pregnancy [13,14]. However, due to various leukocyte subpopulations and different leukocyte phenotypes, it is quite challenging to understand the immune mechanism of fetal acceptance [15]. Natural killer (NK) cells are vastly investigated among decidual cells, mostly responsible for cytotoxic properties as the response to maternal infection and are proven to be increased in PE pregnancies. Therefore, NK cells receive much more attention than CD8^+^ T cells, which are more prominent leukocytes in decidua basalis especially in the late gestational phase [16]. Numerous studies suggest the significance of CD8^+^ T cells in normal pregnancies [17,18,19,20,21].

There are four major subpopulations of CD8^+^ T cells regarding of their main properties and expression of specific markers (naïve, central memory (CM), effector memory (EM), and effector) [22]. Effector and EM CD8^+^ T cell subpopulations are mainly cytotoxic, expressing PRF1, GZMA, GzB, and GNLY that are essential for their cytotoxic capacity. GzB is an enzyme that cleaves and thus activates caspase enzymes present in the cytoplasm of the target cell, whose function is to initiate programmed cell death after activation. PRF1 is required for the delivery of granzyme to the cytoplasm of the target cell [23]. GNLY is a specific protein that, besides cytotoxic activity, serves as a distinctive biomarker of cell-mediated immunity, tumor immunity, infection, and graft versus host disease (GVHD) [24]. GZMA is a cytotoxic protein appearing as alternative cytotoxic path and it is mainly expressed in EM T cells [25,26].

In healthy pregnancies, the majority of the mPBL CD8^+^ T cells are classified as naïve, effector, and EM, while decidual CD8^+^ T cells are mostly EM and, unlike mPBL CD8 effector cells, show decreased expression of PRF1 and GzB [27]. Compared to peripheral blood, decidual surface contains more highly differentiated CD8^+^ than CD4^+^ lymphocytes that suggests strong feto-placental antigen stimulation of these cells [28,29,30,31]. Effector CD8^+^ T cells are crucial in enabling balance between tolerance of the feto-placental unit and involvement in the immune response due to infections. However, they are also thought to participate in the immune tolerance that is crucial for normal pregnancy [32,33]. Some previous studies revealed increased GNLY expression in serum of women with PE, while in decidua it was mostly expressed in NK cells [34,35]. It was found that the proportion of memory CD8^+^cells was decreased in PE pregnancies compared to the healthy group, but without data on the presence of cytotoxic proteins [36].

A major problem with analyzing immune cells in pregnancy is their dynamics; there are some variations in their number during pregnancy [37] and a gestational-age matched control group is required for appropriate interpretation of the results.

Based on our previous research, we wanted to further explore the subpopulations of decidual cytotoxic CD8^+^ T cells and compare it to mRNA expression of cytotoxic proteins in CD8^+^ T cells in mPBL. We analyzed tissue of decidua basalis and peripheral blood of pregnancies complicated with severe and mild PE and compared it to healthy gestational age-matched control pregnancies.

## 2. Materials and Methods

### 2.1. Tissue Preparation and Clinical Data

Material for the analysis was placental tissue, including 10 placentas from women with severe PE, 10 placentas with mild PE, and 20 healthy gestational age-matched placentas as a control. Control 1 (*n* = 10) refers to the healthy gestational age-matched control group for severe PE, and Control 2 (*n* = 10) to the control group for mild PE (Table 1).

The including criteria were singleton pregnancy and confirmed diagnosis of severe or mild PE, defined by American College of Obstetricians and Gynecologists (ACOG) as elevated blood pressure with proteinuria or signs of other organs’ damage. Elevated blood pressure refers to ≥140 mmHg for systolic and ≥90 mmHg for diastolic pressure measured twice within 4 h in previously normotensive woman. Without the presence of proteinuria, PE can be defined with the occurrence of one or more of the following parameters: thrombocytopenia, progressive renal failure, liver damage, pulmonary edema, and cerebral and visual impairment. Severe PE is defined as newly established gestational hypertension with blood pressure ≥ 160/110 mmHg, measured twice in a time interval of 4 h with possible damage of the other organs mentioned above. Mild PE is considered to be the systolic pressure < 160 mmHg and diastolic < 110 mmHg [6]. Clinical features including the maternal age, body mass index (BMI), gestational age, complications, and pregnancy outcomes were also collected, analyzed, and compared among investigated groups (Table 1).

The exclusion criteria in all groups were history of hypertension, diabetes mellitus type 1 or 2, chorioamnionitis, any inflammatory disease, multiple gestations, and assisted reproduction methods. Most patients with severe PE completed pregnancy by the 35th week, unlike mild PE where the mean age of pregnancy termination was the 38th week.

Placentas were collected upon vaginal or Cesarean section deliveries. Placental tissue samples were fixed in 4% formalin before the tissue processing. Tissue samples containing basal decidua (1 × 1 cm^2^ in size) were cut, washed in phosphate buffer, dehydrated in alcohol, purified in xylol, and then embedded in paraffin. Tissue sections were cut at 4µm on rotatory microtome and mounted on silanized slides [38]. Placental tissue was macroscopally normal in all investigated groups.

### 2.2. Double Immunofluorescence Staining

Tissue sections were deparaffinized in xylol, which was followed by rehydration through descending concentrations of alcohol and twice through distilled water. Antigen retrieval was performed by heating (via microwave oven) the sections in citrate buffer pH6, pH9, or ethylene diamine tetra acetic acid (EDTA) buffer for 17 min. After cooling to room temperature, tissue sections were incubated with the combination of primary antibodies (Supplemental Table S1) for 1 h. Sections were then washed in PBS and incubated with the proper combination of secondary antibodies: goat anti-mouse rhodamine (1:300 AP124R; Jackson Immuno Research Lab, West Grove, PA, USA) and goat anti-rabbit FITC (1:300 AP132F; Jackson Immuno Research Lab, West Grove, PA, USA) in PBS for 1 h or anti-mouse IgG (H+L), F(ab’)2 Fragment Alexa Fluor^®^ 488 Conjugate (1:500 4408S; Cell Signaling Technology Inc, Danvers, MA, USA) in PBS and anti-rabbit IgG (H+L), F(ab’)2 Fragment Alexa Fluor^®^ 594 Conjugate (1:500 8889as; Cell Signaling Technology Inc, Danvers, MA, USA) in PBS for 1 h. After the incubation period, sections were rinsed in PBS, counterstained with DAPI and covered with coverslip (Immuno-mount, Shandon Inc., Pittsburgh, PA, USA). The number of stained GNLY^+^, GNLY^+^CD8^+^, GzB^+^, GzB^+^CD8^+^, PRF1^+^, and PRF1^+^CD8^+^ cells was counted in placental decidua basalis. All the tissue sections were examined using a ×40 objective on Olympus BX51 (Olympus, Tokyo, Japan) and photographed with DP71 camera (Olympus, Tokyo, Japan). Ten areas were used for the analysis of a minimum 1 mm^2^ of decidual tissue. The empty spaces were excluded from the analysis. Negative control tissue was prepared following the same protocol, except PBS was used for the incubation instead of primary antibodies. Lymph node tissue was used as a positive control. All sections were analyzed by two independent observers (VS and MB) in a blinded manner.

### 2.3. Isolation of CD8^+^ T Cells from mPBL

We took 10 mL of peripheral blood in the test tube containing EDTA for the analysis. CD8^+^ T cells of women with PE and normal pregnancies were isolated (from peripheral blood) with EasySepTMHuman CD8 Positive Selection Kit II (18053, StemCell Technologies, Vancouver, Canada). We used Lymphoprep™ (07801, StemCell Technologies, Vancouver, Canada) as the recommended medium for the isolation of the mononuclear cells from peripheral blood. Afterwards, we isolated mononuclear cells from the blood sample, counted them, checked the total number which had to be higher than 1 × 10^8^/mL in the volume of 0.1 to 2.5 mL. This was followed by the isolation of CD8^+^ T cells. Purity of the selected CD8^+^ T cells was checked by flow cytometry. The resulting CD8^+^ T cells were > 98% as determined by immunofluorescence analysis with directly labelled mAb. CD8^+^ T cells were used for RNA isolation. Total RNA was extracted from the CD8^+^ T cells by QIAamp RNA Blood Mini kit (QI52304, Qiagen, Germantown, MD, USA) according to the manufacturer’s instruction. RNA concentrations were determined by Quibit (Q33327, Thermo Scientific, Waltham, MA, USA). Total RNA extracted from CD8^+^ T cells was used for RT-PCR and qPCR analysis.

### 2.4. Flow Cytometry Analysis

Two mL whole blood was collected using the anticoagulant tubes of EDTA and 100 µL whole blood was stained for surface markers with CCR7-FITC, CD28-PE, CD45RA-PE-Cy™7, CD27-PerCP-Cy™5.5, CD8-APC, and CD3-APC-H7 obtained from BD Biosciences.

Cells were acquired on CANTO II flow cytometer (BD Biosciences, Franklin Lakes, NJ, USA) where at least 100,000 events in lymphocyte scatter gate were acquired. Staining specificity was confirmed using fluorescence minus one (FMO) and all antibodies minus one. Data analysis was carried out using BD FACSDiva software™ (BD Biosciences, Franklin Lakes, NJ, USA).

### 2.5. RT-PCR

Two nanograms of total RNA were reverse transcribed into complementary DNA (cDNA) with a High Capacity Reverse Transcriptase Kit (Applied Biosystems, Foster City, CA, USA) using random primers according to the manufacturer’s instructions. cDNA (final volume of 20 µL) was stored at −80 °C for subsequent quantification gene of interest. PCR was performed using AmpliTaq Gold PCR Master Mix (Applied Biosystems Waltham, MA, USA). Sequences of selected primers (Sigma Aldrich, Sant Louis, MI, USA) and the respective product lengths are shown in Supplemental Table S2. Optimal annealing temperature was evaluated for the positive selection CD8^+^ T cells. Nuclease-free water containing no cDNA was used as negative control in each experiment. The PCR products stained with EtBr or Sybr green solution were proceed to electrophoresis using 2% agarose gel and exposed to UV light for visualization.

### 2.6. qPCR

qPCR analysis was performed on Real-Time PCR instrument (Applied BiosystemsFast 7500, Waltham, MA, USA) using Taqman^®^ Fast Advanced Universal Master Mix II (Applied Biosystems, Waltham, MA, USA) containing AmpEraseuracil-N-glycosylase and the passive reference dye ROX. ProbesTaqman^®^ gene expression assays for human GZMA, GzB, GNLY, PRF1, and FOXP3 were supplied by Applied Biosystems (Hs00196206_m1, Hs00188051_m1, Hs01120098_g1, Hs00169473_m1, and Hs00203958_m1). Housekeeping genes 18sRNA (Hs03003631_g1) and glyceraldehyde-3-phosphate dehydrogenase (GAPDH; Hs02758991_g1) were analyzed. GAPDH mRNA expression was normally distributed showing lower standard deviation in comparison to mRNA expression of 18sRNA. Therefore, GAPDH was selected as a housekeeping gene. Taqman real-time PCR was performed using approximately 50 ng cDNA template, 1 μLTaqman^®^ (Applied Biosystems, Waltham, MA, USA) gene expression assay and 10 μLTaqman^®^ (Applied Biosystems, Waltham, MA, USA) universal master mix (20 μL of final volume). The PCR protocol used involved heating for 2 min at 50 °C for uracil-N-glycosylase activation, then heating for 2 min at 95 °C for polymerase activation, followed by 40 cycles of amplification (3 s at 95 °C and 30 s at 60 °C). We performed triplicate PCRs per gene, per cDNA sample. A negative control containing nuclease-free water instead of cDNA template was used in each experiment. The ^2−ΔΔ^CT method was used as the method of relative quantification.

### 2.7. Statistical Analysis

Statistical analysis (Kruskal–Wallis and Dunn’s post hoc tests) was performed with Prism8.00 for Windows (GraphPad software, San Diego, CA, USA). Data were presented as median ± IqR. *p* < 0.05 was set as significant.

## 3. Results

### 3.1. Quantification of Cytotoxic CD8^+^ T Cells in Decidua Basalis of Severe and Mild PE Compared to Normal Healthy Pregnancies

**Figure 1:**
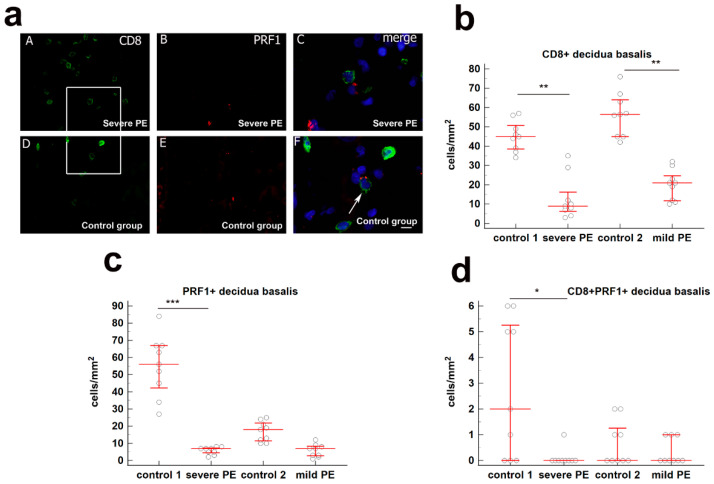
(**a**) (**A**–**C**) Co-expression of CD8 and PRF1 markers in decdua basalis cells of the third trimester pregnancies in severe PE. Double immunofluorescence staining showed CD8 (**A**) and PRF1 (**B**) positive cells. Merging (**A**) and (**B**) revealed no co-expression of CD8 and PRF1 (**C**). Scale bar = 10 µm. (**D**–**F**) Co-expression of CD8 and PRF1 markers in decdua basalis cells of the third trimester pregnancies in control group. Double immunofluorescence staining showed CD8 (**D**) and PRF1 € positive cells. Merging (**D**) and (**E**) revealed co-expression of CD8 and PRF1 (arrow) (**F**). Scale bar = 10 µm. Magnification frame on (**A**) and (**D**) is shown on (**C**) and (**F**), respectively; magnification on (**A**,**B**,**D**,**E**) is ×40; magnification on (**C**) and (**F**) is ×80. Expression of (**b**) CD8^+^, (**c**) PRF1^+^, and (**d**) CD8^+^PRF1^+^ in decidua basalis in severe PE (*n* = 9), mild PE (*n* = 9) and healthy age-matched control 1 (*n* = 9) and control 2 (*n* = 9). Data are presented as the median ± interquartile range (vertical line). Significant differences were indicated by *p* * < 0.01, *p* ** < 0.001, *p* *** < 0.0001 (Kruskal–Wallis test followed by Dunn’s multiple comparison test).

With the purpose to determine the localization of CD8^+^GNLY^+^, CD8^+^GzB^+^, and CD8^+^ PRF1^+^ cells in decidua basalis, we analyzed paraffin-embedded placental tissue containing decidua basalis of both, PE and gestational age-matched control groups. A high variation in the numbers of CD8^+^ T cells was observed between the severe PE and mild PE group compared to gestational age-matched control groups (*p* < 0.001; *p* < 0.001; Figure 1b).

The total number of PRF1^+^ cells/mm^2^ (median = 7; IqR = 4–7.5; median = 56; IqR = 39.5–67, respectively), and CD8^+^PRF1^+^ cells/mm^2^ (median = 2; IqR 0–5.5 median = 0; IqR 0–0, respectively) in decidua basalis of severe PE was significantly lower in comparison to the gestational age-matched control group (*p* < 0.0001; *p* < 0.01). The difference between the PRF1^+^ cells/mm^2^ and CD8^+^PRF1^+^ cells/mm^2^ in decidua basalis of mild PE and gestational age-matched control groups was not significant. These results are graphically summarized in Figure 1a,c,d.

**Figure 2:**
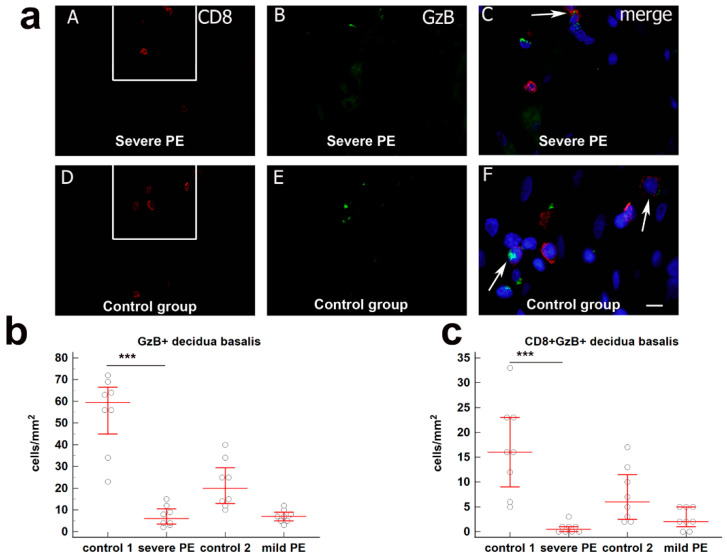
(**a**) (**A**–**C**) Co-expression of CD8 and GzB markers in decdua basalis cells of the third trimester pregnancies in severe PE. Double immunofluorescence staining showed CD8 (**A**) and GzB (**B**) positive cells. Merging (**A**) and (**B**) revealed co-expression of CD8 and GzB (arrow) (**C**). Scale bar = 10 µm. (**D**–**F**) Co-expression of CD8 and GzB markers in decdua basalis cells of the third trimester pregnancies in control group. Double immunofluorescence staining showed CD8 (**D**) and GzB (**E**) positive cells. Merging (**D**) and (**E**) revealed co-expression of CD8 and GzB (arrows) (**F**). Scale bar = 10 µm. Magnification frame on (**A**) and (**D**) is shown on (**C**) and (**F**), respectively; magnification on (**A**,**B**,**D**,**E**) is ×40; magnification on (**C**) and (**F**) is ×80. Expression of (**b**) GzB+ and (**c**) CD8+GzB+ in decidua basalis in severe PE (*n* = 8), mild PE (*n* = 8) and healthy age-matched control 1 (*n* = 8) and control 2 (*n* = 8). Data were presented as the median ± IqR (vertical line). Significant differences were indicated by *p* *** < 0.0001 (Kruskal–Wallis test and Dunn’s multiple comparison test).

The total number GzB^+^ cells/mm^2^ (median = 59.5; IqR = 39.5–67.75, median = 6; IqR 3.25–11.25) and CD8^+^GzB^+^ cells/mm^2^ (median = 16; IqR = 7.5–23; median = 0.5; IqR 0–1) in decidua basalis of severe PE was significantly decreased compared to the gestational age-matched control group (*p* < 0.0001, respectively). The difference between the GzB cells/mm^2^ and CD8^+^GzB^+^ cells/mm^2^ in decidua basalis of mild PE and gestational age-matched control groups was not statistically significant. These results are graphically summarized in Figure 2.

**Figure 3:**
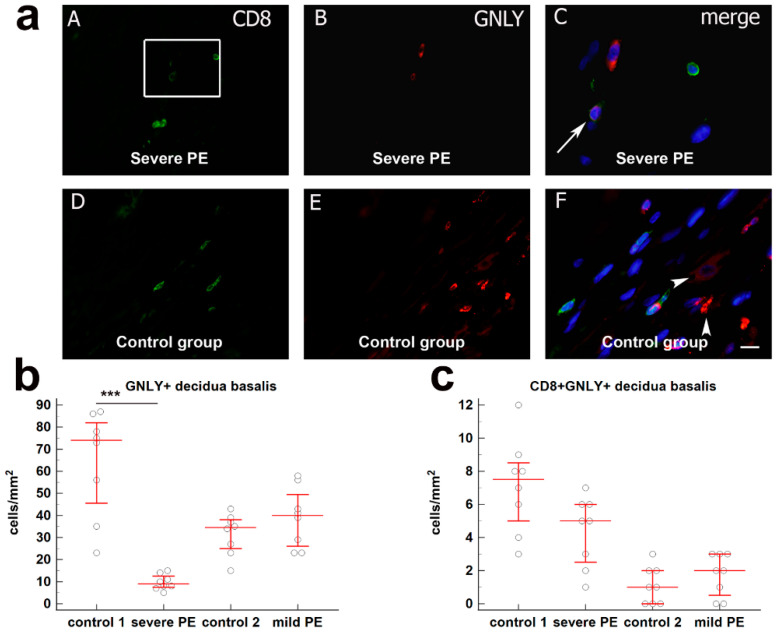
(**a**) (**A**–**C**) Co-expression of CD8 and GNLY markers in decidua basalis cells of the third trimester pregnancies in severe PE. Double immunofluorescence staining showed CD8 (**A**) and GNLY (**B**) positive cells. Merging (**A**,**B**) revealed co-expression of CD8 and GNLY (arrow) (**C**). (**D**–**F**) Co-expression of CD8 and GNLY markers in decidua basalis cells of the third trimester pregnancies in control group. Double immunofluorescence staining showed CD8 (**D**) and GNLY (**E**) positive cells. Merging (**D**) € revealed co-expression of CD8 and GNLY in T cells, and granular or diffuse expression of GNLY in NK and EVT cells (arrowheads) (**F**). Magnification frame on (**A**) is shown on (**C**); magnification on (**A**–**E**) is ×40; magnification on (F) is ×80; Scale bar = 10 μm. Expression of (**b**) GNLY+ and (**c**) CD8+GNLY+ in decidua basalis in severe PE (*n* = 8), mild PE (*n* = 8) and healthy age—matched control 1 (*n* = 8) and control 2 (*n* = 8). Data were presented as the median ± IqR (vertical line). Significant differences were indicated by *p* *** < 0.0001 (Kruskal–Wallis test and Dunn’s multiple comparison test).

The total number GNLY^+^ cells/mm^2^ in decidua basalis of severe PE (median = 74; IqR = 40.25–84; median = 9; IqR 7.25–13.25) was significantly decreased compared to the gestational age-matched control group (*p* < 0.0001). The difference between GNLY^+^ cells/mm^2^ in mild PE and gestational age-matched control groups as well as the difference for the CD8^+^GNLY^+^ expression, were not statistically significant in all the examined groups. These results are graphically summarized in Figure 3.

### 3.2. Quantification of Cytotoxic Proteins PRF1, GNLY, GZMA, GzB, and FOXP3 in CD8^+^ T Cells from mPBL of Severe and Mild Preeclampsia Compared to Normal Healthy Pregnancies

**Figure 4:**
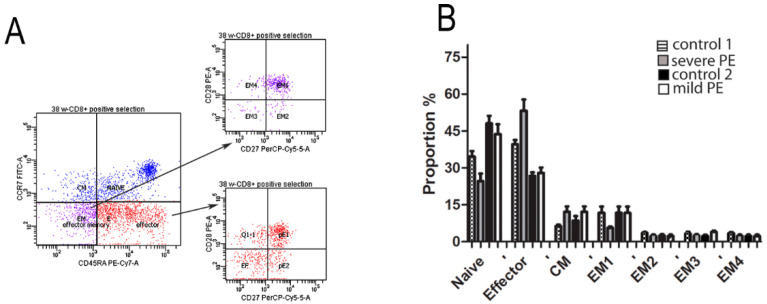
(**A**) Differential expression of CD45RA, CCR7, CD27, and CD28 cell surface markers on total CD8^+^ T cells from maternal peripheral blood control. CD3^+^CD8^+^ gated cells were separated into four subsets (naive, CM, EM, and effector) based on CD45RA and CCR7 labelling. Blue color presents naïve and CM CD8^+^ T cells. Effector (red) and EM (purple) were analyzed for CD27 and CD28 co-expression, and the proportion of effector (eP1 and eP2) and EM (EM1, EM2, EM3, and EM4) cells were determined. (**B**) The proportion of CD8 naïve, CM, effector, and EM (EM1, EM2, EM3, and EM4) cells is determined in mPBL from control 1 (*n* = 10), severe PE (*n* = 10), control 2 (*n* = 10), and mild PE (*n* = 10).

Based on flow cytometry, we classified CD8^+^ T cells into four functionally different populations: naïve (RA^+^CCR7^+^), effector (RA^+^CCR7^−^), CM (RA^−^CCR7^+^), and EM (RA^−^CCR7^+^). Further analysis revealed four subsets of EM cells: EM1 (CD28^+^CD27^+^), EM2 (CD28^−^CD27^+^), EM3 (CD28^−^CD27^−^), and EM4 (CD28^+^CD27^−^) and three subsets of effector cells: pre-effector 1 (PE-1; CD28^+^CD27^+^), pre-effector 2 (PE-2; CD28^−^CD27^+^), and effector cells (CD28^−^CD27^−^). Flow cytometry analysis revealed that majority of mPBL CD8^+^ T cells from PE and healthy pregnancies were naïve, effector, and EM1, but without significant difference among investigated groups (Figure 4).

**Figure 5:**
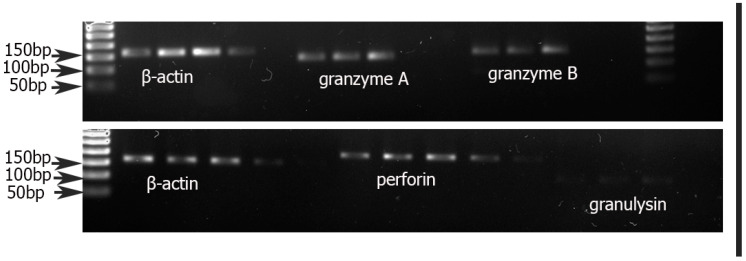
Agarose gel electrophoresis of RT-PCR products from granzyme A (135 bp), granzyme B (149 bp), perforin (200 bp), granulysin (123 bp), and β-actin (161 bp) mRNA of CD8^+^T cells from maternal peripheral blood.

RT-PCR for PRF1, GZMA, GzB, and GNLY mRNA was performed from total RNA isolated from positively selected CD8^+^ T cells. mRNA for all cytotoxic proteins mentioned above were identified in all samples (Figure 5).

**Figure 6:**
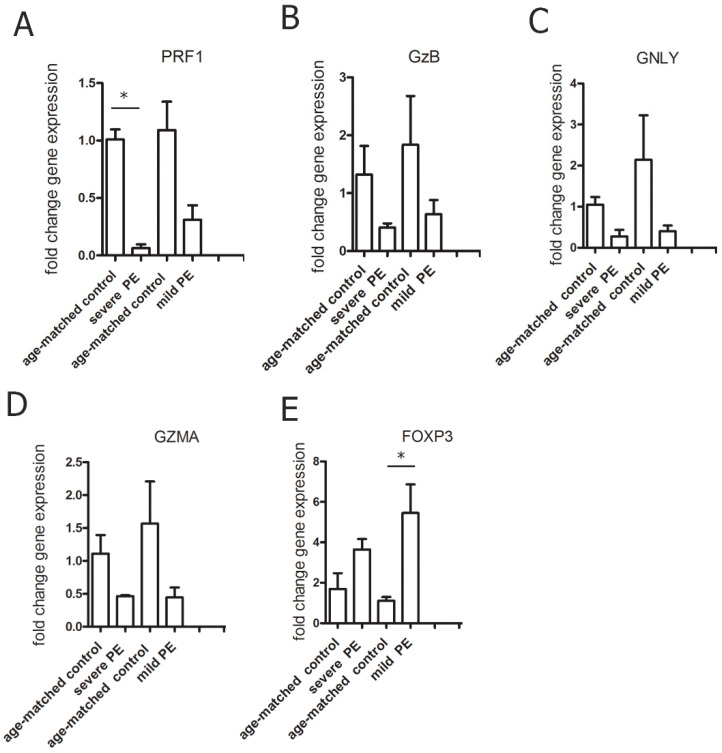
qPCR mRNA fold change of (**A**) PRF1, (**B**) GzB, (**C**) GNLY, (**D**) GZMA, and (**E**) FOXP3 in CD8^+^ T cells from peripheral blood of severe PE (*n* = 8) and mild PE (*n* = 8) compared to age-matched control 1 (*n* = 8) control 2 (*n* = 8). Significant differences are indicated by * *p* < 0.01 (Kruskal–Wallis test and Dunn’s multiple comparison test).

We found mRNA expression of PRF1 significantly downregulated in severe PE (*p* < 0.01) while FOXP3 mRNA expression was upregulated in mild PE (*p* < 0.01) compared to gestational age-matched healthy controls. We noticed that mRNA expression of other cytotoxic proteins GNLY, GZMA, and GzB in CD8^+^ T cells of mPBL trend toward lower expression (Figure 6).

## 4. Discussion

Numerous studies suggest an important role of CD8^+^ T cells in normal pregnancies [17,18,19]. In the menstrual cycle of human endometrium, CD8^+^ T cells have cytotoxic ability, which is significantly low during the secretory phase. This might indicate a potential role of CD8^+^ T cells in accepting the conceptus [17]. An increased number of CD8^+^ T cells was observed at the time of implantation and, in contrast, a significantly decreased number was found in endometrial tissue of women suffering infertility of unknown origin [39]. The study of Scaife et al. demonstrated that cytokine production and cytotoxic capacity of CD8^+^ T cells controls trophoblast invasion [19,40].

However, data regarding the phenotype and function of mPBL and decidual CD8^+^ T cells during pregnancy is much more limited. Tilburgs et al. showed a significant increase in decidual CD8^+^ T cells compared to peripheral blood in healthy pregnancies. Their research describes highly differentiated CD8^+^ T cells on the fetal-maternal surface of normal pregnancies [27]. Similar to their research, in the mPBL of PE pregnancies, we showed that most of CD8^+^ T cells subpopulations were naïve, effector, and EM. However, with further analysis of CD8^+^ T cells in women with severe PE, we noticed downregulation in mRNA expression of cytotoxic granules PRF1, GZMA, GzB, and GNLY. We can assume that these findings were the result of a decreased number of cytotoxic effector CD8^+^ T cells.

FOXP3 mRNA expression in CD8^+^ T cells was upregulated in our study, which was surprising as the results of numerous recent studies emphasize the role of regulatory CD4^+^CD25^+^FOXP3^+^ T cells for fetal acceptance [41,42,43,44,45,46]. The CD8^+^ FOXP3^+^ Treg subtype accounts for a small percentage of Treg cells and is much less studied compared to CD4^+^FOXP3^+^ subpopulation. Similar to conventional T regulatory cells (CD4^+^CD25^+^FOXP3^+^) CD8^+^FOXP3^+^ cells are considered to be immunosuppressive and are associated with immune suppression in human and mouse subjects. The difficulty to differentiate CD8^+^ Tregs from conventional CD8^+^ T cells resulted in dissatisfactory characterization of phenotype and function of these cells [47,48,49]. Our future study aims to investigate CD8^+^FOXP3^+^ in more detail considering their immunosuppressive potential. The question arises whether decreased number of these cells could be associated with pregnancy failure and PE. Elevated FOXP3 expression in CD8^+^ T cells in mPBL may be explained as an attempt to suppress exacerbated inflammatory response present in PE [50].

Decidual CD8^+^ T cells have been investigated in normal, healthy, early and late pregnancy, and studies have emphasized the importance of these cells for pregnancy maintenance [39,51,52]. Scaife et al. analyzed decidual samples (*n* = 51) of early pregnancy from 7 to 14 weeks of gestation, and showed that CD8^+^ T cells via specific cytokines in vitro facilitate trophoblast invasion in early pregnancy [19]. Decidual distribution and phenotype of CD8^+^ T cells in pregnancies complicated with PE are scarce. Rieger et al. showed a decreased proportion of CD8+ and αβ-T cell receptors, but not γδ-T cells in decidual tissue of pregnancies complicated with PE compared to control group [53]. Some previous papers revealed that decidual CD8^+^ T cells of healthy pregnancy, unlike peripheral blood CD8^+^ T cells, do not express cytotoxic molecules GzB and PRF1 [9,16,27]. Our recent work showed a significantly decreased number of decidual CD8^+^ T cells in placentas of severe PE [38], rather surprising data, as general inflammatory activation is one of the main characteristics of PE [54]. Some other studies are in line with this result. Rieger et al. showed a decreased number of decidual CD8^+^ T cells analyzing 33 placentas of pregnancies complicated with PE, of which 27 were early PE [53]. Another study by Williams et al. on 12 samples of decidual tissue of pregnancies complicated with PE obtained results consistent with our study [55]. The mechanisms of feto-placental immune recognition and effector cell functions of T cells in decidua basalis remain poorly understood.

Confirming our previous study, the total number of decidual CD8^+^ T cells analyzed by immunofluorescence was significantly reduced in the group of severe and mild PE in comparison to normal pregnancy group. GNLY and PRF1 were decreased in decidua of women with PE but, given the number, they represent two main cytolytic molecules. Additionally, we noticed that GNLY is a cytotoxic protein that is, besides in decidual lymphocytes, significantly expressed and visible as diffuse staining in the cytoplasm of EVT cells, which is consistent with other recent studies [56].

The proportion of decidual cytotoxic CD8^+^ T cells containing PRF1 and GzB was significantly reduced, but not the proportion of those containing GNLY. Decreased cytotoxic CD8^+^ T cells were observed only in severe PE compared to normal pregnancy group. These data imply that decidual and peripheral blood CD8^+^ T cells of pregnancies complicated with severe PE may have decreased cytotoxic function. However, the dynamic experiments of cytotoxic activity of decidual CD8^+^ T cells would provide some more clarity to establish the role of decidual CD8^+^ T cells in pathophysiology of PE. Maternal placental lymphocytes isolated in vitro after 34 weeks of gestation could contain fetal lymphocytes originating from chorionic villi capillaries. Therefore, we cannot be completely sure that we have an isolated population of decidual CD8^+^ T cells. The main reason is that the decidua is so thin that, macroscopically or microscopically, it cannot be completely separated from the chorionic villi. In preeclampsia, decidua basalis is not properly developed, and it is not well “recognized” by trophoblast. Hence, the separation is even more difficult. Additionally, there is no particular marker that can distinguish maternal from fetal decidual CD8^+^ T cells. The results, in addition to our previous research, show that decidua basalis of women with PE expresses a significantly decreased number of CD25^+^FOXP3^+^ cells and activated T cells (CD4^+^CD25^+^), as well as a reduced overall number of cytotoxic CD8^+^ T cells. These results may be due to a decrease in total CD8^+^ T cell count, but also to a systemic maternal response, as the mRNA expression of cytotoxic granules in mPBL CD8^+^ T cells was downregulated and FOXP3 upregulated.

The major limitation of our study that may have affected the results was the time of placental tissue examination and the different mode of delivery between severe PE and control group. Placentas were collected immediately after delivery, and there are usually 3 days until immunofluorescence examination. This period is necessary for the correct preparation of tissue and it cannot be avoided. The mode of delivery could affect the number of immune cells. Previous studies reported disproportion in the number of T cells between vaginal delivery and Cesarean section and this should be taken into account [57]. However, the study of van Egmond et al. is encouraging on this issue, as they did not find differences in the number of CD8^+^ T cells in mPBL before and after elective Cesarean delivery [58]. Additionally, although sample size was sufficient to conduct the study, more of samples would provide more accurate results.

## 5. Conclusions

We showed that decidual cytotoxic CD8^+^ T cells are decreased in pregnancies complicated with PE, with additionally decreased expression of cytotoxic proteins PRF1, GzB, and GNLY. However, additional dynamic experiments should be conducted to clarify the role of cytotoxic CD8^+^ T cells in the development of PE. In contrast to some previous findings, FOXP3 mRNA expression in mPBL CD8^+^ T cells was upregulated. Therefore, in our future work, we want to investigate the presence of CD8^+^FOXP3^+^ cells in the decidua basalis and peripheral blood of women with PE. This would further contribute to define the subpopulations of T regulatory cells that certainly play the most important role in pathophysiology of PE.

## Figures and Tables

**Table 1:** Clinical features of the patients in the study groups.

	Control 1 (*n* = 10)	Severe Preeclampsia (*n* = 10)	Control 2 (*n* = 10)	Mild Preeclampsia (*n* = 10)	*p*-Value
Maternal age (years) mean ± SD	29.08 ± 5.81	27.50 ± 4.51	28.42 ± 5.22	31.30 ± 5.73	0.390
Gestational age (weeks) median (IqR)	35 (34–39)	34 (33–39)	39 (38–41)	38 (34–41)	<0.001
Systolic RR (mmHg) mean ± SD	118 ± 5.23	173 ± 18.07	113 ± 5.72	141 ± 7.35	<0.001
Diastolic RR (mmHg) median (IqR)	75 (66–81)	121 (111–140)	77 (65–80)	89 (86–100)	<0.001
Birth weight (grams) median (IqR)	2340 (2123–3220)	1820 (1250–2200)	3310 (2370–4500)	3315 (1803–3950)	<0.001
Cesarean deliveries (%)	2 (20)	10 (100)	1 (10)	2 (20)	<0.001
Body mass index (BMI) mean ± SD	24.94 ± 4.86	23.67 ± 2.42	25.84 ± 4.16	23.52 ± 2.16	0.06
Intrauterin growth restriction (IUGR) (%)	1 (10)	7 (70)	0 (0)	1 (10)	<0.001
Postpartal complications (%)	0 (0)	6 (60)	0 (0)	1 (10)	<0.001

Data are presented as mean ± SD analyzed by ANOVA, median (IqR = interquartile range) analyzed by Kruskal–Wallis test, and percentage using Chi-square test.

## Data Availability

The datasets used and/or analyzed during the current study are available from the corresponding author on request.

## Supplementary Materials

The following are available online at https://www.mdpi.com/article/10.3390/biology10101037/s1, Table S1. Primary antibodies used for double immunofluorescence staining. Table S2. Primer sequences of selected target genes.
