# Supplementary material for: Decreased Expression of Cytotoxic Proteins in Decidual CD8+ T Cells in Preeclampsia
Source: Biology (Basel). 2021 Oct 13;10(10):1037. doi: 10.3390/biology10101037 (PMC8533461; doi:10.3390/biology10101037)
Supplement: Supplementary file 1 [file biology-10-01037-s001.zip › biology-1383522-supplementary.pdf]

Supplementary Table S1. Primary antibodies used for double immunofluorescence staining.

| Antibody | Dilution | Host | Cellular localization | Developer |
|---|---|---|---|---|
| **CD8** | 1:100 | Mouse | Membrane | Dako M7103 |
| **CD8** | 1:100 | Rabbit | Membrane | Sigma SAB5500074 |
| **Perforin** | 1:200 | Rabbit | Cytoplasm | Cell Signaling 31647S |
| **Granulysin Anti-GNLY** | 1:1000 | Rabbit | Cytoplasm | Sigma HPA058021 |
| **Granzyme B** | 1:20 | Mouse | Cytoplasm | Sigma MAB3070 |

Supplementary Table S2. Primer sequences of selected target genes.

| Gene | Forward primer | Reverse primer | Amplicon (bp) |
|---|---|---|---|
| **Beta actin** | ACCACACCTTCTACAATGAG | TAGCACAGCCTGGATAGC | 161 |
| **Granzyme B** | CTGATACGAGACGACTTC | GGATTATAGGCTGGATGG | 149 |
| **Granzyme A** | ATGGTCCTACTTAGTCTTG | CCTGGTTATTGAGTGAGC | 135 |
| **Perforin** | GGTTCACTGCCACGGATG | ACAGGTGCCAAGGAGGTC | 200 |
| **Granulysin** | GAAGAAGATGGTGGATAAG | CTAGACTGATACCTCCTC | 123 |
